# Whole plant chamber to examine sensitivity of cereal gas exchange to changes in evaporative demand

**DOI:** 10.1186/s13007-018-0357-9

**Published:** 2018-11-01

**Authors:** Iván Jauregui, Shane A. Rothwell, Samuel H. Taylor, Martin A. J. Parry, Elizabete Carmo-Silva, Ian C. Dodd

**Affiliations:** 1 Lancaster Environment Centre, Lancaster University, Lancaster, LA1 4YQ UK; 2 Present Address: Plant Genetics, TERRA Teaching and Research Center, Gembloux Agro Bio-Tech, University of Liège, Gembloux, 5030 Belgium

**Keywords:** Photosynthesis, Transpiration, Water use efficiency, VPD, ABA

## Abstract

**Background:**

Improving plant water use efficiency (WUE) is a major target for improving crop yield resilience to adverse climate change. Identifying genetic variation in WUE usually relies on instantaneous measurements of photosynthesis (An) and transpiration (Tr), or integrative measurements of carbon isotope discrimination, at the leaf level. However, leaf gas exchange measurements alone do not adequately represent whole plant responses, especially if evaporative demand around the plant changes.

**Results:**

Here we describe a whole plant gas exchange system that can rapidly alter evaporative demand when measuring An, Tr and intrinsic WUE (iWUE) and identify genetic variation in this response. An was not limited by VPD under steady-state conditions but some wheat cultivars restricted Tr under high evaporative demand, thereby improving iWUE. These changes may be ABA-dependent, since the barley ABA-deficient mutant (*Az34*) failed to restrict Tr under high evaporative demand. Despite higher Tr, *Az34* showed lower An than wild-type (WT) barley because of limitations in Rubisco carboxylation activity. Tr and An of *Az34* were more sensitive than WT barley to exogenous spraying with ABA, which restricted photosynthesis via substrate limitation and decreasing Rubisco activation.

**Conclusions:**

Examining whole plant gas exchange responses to altered VPD can identify genetic variation in whole plant iWUE, and facilitate an understanding of the underlying mechanism(s).

**Electronic supplementary material:**

The online version of this article (10.1186/s13007-018-0357-9) contains supplementary material, which is available to authorized users.

## Background

Photosynthesis is a complex process in which light, water and carbon dioxide (CO_2_) are used to synthesize carbohydrates. In plants, CO_2_ can only diffuse into the leaves via the stomata. When open, the stomata represent the major path of water loss to the atmosphere via transpiration. Approximately 98% of all water taken up through the roots may be transpired through the stomata [1]. Therefore, plants constantly seek to minimise water loss while maintaining CO_2_ entry for photosynthesis, by tightly regulating their stomatal responses. Monitoring plant–atmosphere gas exchange is essential for understanding plant responses to a fluctuating environment.

Atmospheric vapour pressure deficit (VPD) or evaporative demand is influenced by both air temperature and relative humidity (RH), and is the difference between the saturation vapour pressure and the actual vapour pressure. The driving force for water movement through the plant is caused by the vapour pressure deficit between the substomatal cavity and the surrounding air, known as leaf-to-air vapour pressure deficit (VPD_leaf_). High VPD_leaf_ increases plant transpiration rates (Tr) [2]. By decreasing their stomatal conductance (gs), plants can partially limit Tr and the decrease in leaf water status [3]. High ambient VPD and VPD_leaf_ enhances evaporation of water from the leaf, reducing bulk leaf water status and inducing stomatal closure, which is contributed to by a hydropassive response common to all land plants and, in angiosperms, a hydroactive response regulated by abscisic acid (ABA) [4]. Increased VPD rapidly upregulates expression of the *NCED* genes (involved in ABA biosynthesis), thereby increasing leaf [ABA] and decreasing gs [5]. However, this leaf-based mechanism may not completely explain the spatial and temporal behaviour of whole plant transpiration under increasing evaporative demand: other factors such as patchy stomatal closure [6], changes in leaf [7], root [8, 9], or whole plant hydraulic conductivity [10, 11] and leaf-age differences in sensitivity to ABA [12], may operate together to limit Tr under increasing VPD.

Water use efficiency (WUE) typically refers to the ratio between the biomass produced and cumulative water use. At the physiological level, the ratio of net photosynthesis (An) to Tr is known as photosynthetic or intrinsic WUE (iWUE). Maintaining net photosynthesis (An) while reducing Tr under high atmospheric evaporative demand may be of adaptive significance under certain environmental conditions, and genetic variability in the sensitivity of gs to VPD has been described in angiosperms: in some genotypes, Tr increases linearly with increasing VPD, while others restrict Tr at higher VPD. Pioneering work identified the “restricted transpiration” trait [13, 14], and associated low leaf hydraulic conductivity with improved WUE. The trait has been identified in many crops, including cereals [15, 16], using gravimetric methods in chambers [17], greenhouses [18], and the field [19]. A potential drawback of decreasing gs to restrict transpiration under increasing VPD, is that internal CO_2_ concentration (Ci) may decrease, thereby decreasing An via substrate limitation. Field measurements under high VPDs cannot separate effects of VPD on An from effects of high temperature per se. Consistent with this potential limitation, high evaporative demands and temperatures considerably limit leaf level photosynthesis [20, 21]. However, similar measurements at the whole plant level have not been made.

Leaf gas exchange measurements fail to capture whole plant responses since: (1) transpiration inside the leaf cuvette of an infra-red gas analysis system reflects the controls imposed on that environment (i.e. mixing of air to control boundary layer conductance, chosen temperature, choice of light source, leaf area used for measurement, flow rate); (2) leaf measurements cannot adequateliy describe whole plant An due to spatial variation in the light environment of different leaves [22, 23]; (3) naturally occurring microclimates across the plant affect its interaction with the environment. Thus, several chambers have been built to characterize whole plant gas exchange of plants such as Arabidopsis [24–26], shrubs [27–29], or even trees [30], but with limited regulation of environmental conditions inside the chamber. As a consequence, such measurements may be bedeviled by leaks, flow rate fluctuations, overheating of the larger chambers [31], and high humidity/condensation that can cause severe failures of IRGAs [32, 33]. These technical difficulties probably explain why relatively few researchers have built whole plant systems to study transpiration responses to increasing evaporative demand [7, 18, 34, 35].

In the present manuscript, we describe a whole plant gas exchange system to measure An, Tr and iWUE under increasing VPD. We tested whether different cereal genotypes, previously demonstrated to show variation in transpiration response to VPD [16] and variation in leaf-level photosynthesis [36], showed variation in whole plant iWUE as evaporative demand changed. Because higher photosynthetic rates correlate with high yield [36] and stomatal responses to VPD governs diurnal plant transpiration [37], identifying useful genetic variation in iWUE at high VPD will be of interest to plant physiologists and breeders. Our whole plant gas exchange system is relevant to achieving this goal.

Since the role of ABA in regulating stomatal responses to VPD is not completely clear (cf. [35, 38]), we used the whole plant gas exchange system to investigate the responses of an ABA-deficient barley mutant and its wild-type under contrasting VPD, and in response to foliar ABA spraying. Previous observations indicate that exogenous ABA application limits photosynthesis of ABA-deficient plants (*flacca* tomato mutant and Arabidopsis lines) [35, 39], even if the mechanistic interpretation is not clear. Our working hypothesis is that stomatal hypersensitivity of the ABA deficient mutant (*Az34*) to exogenous ABA spraying constrains photosynthesis via substrate limitation, decreasing Rubisco activation state, and limiting net photosynthesis.

## Materia methods

### Growth conditions and plant material

**Fig. 1:**
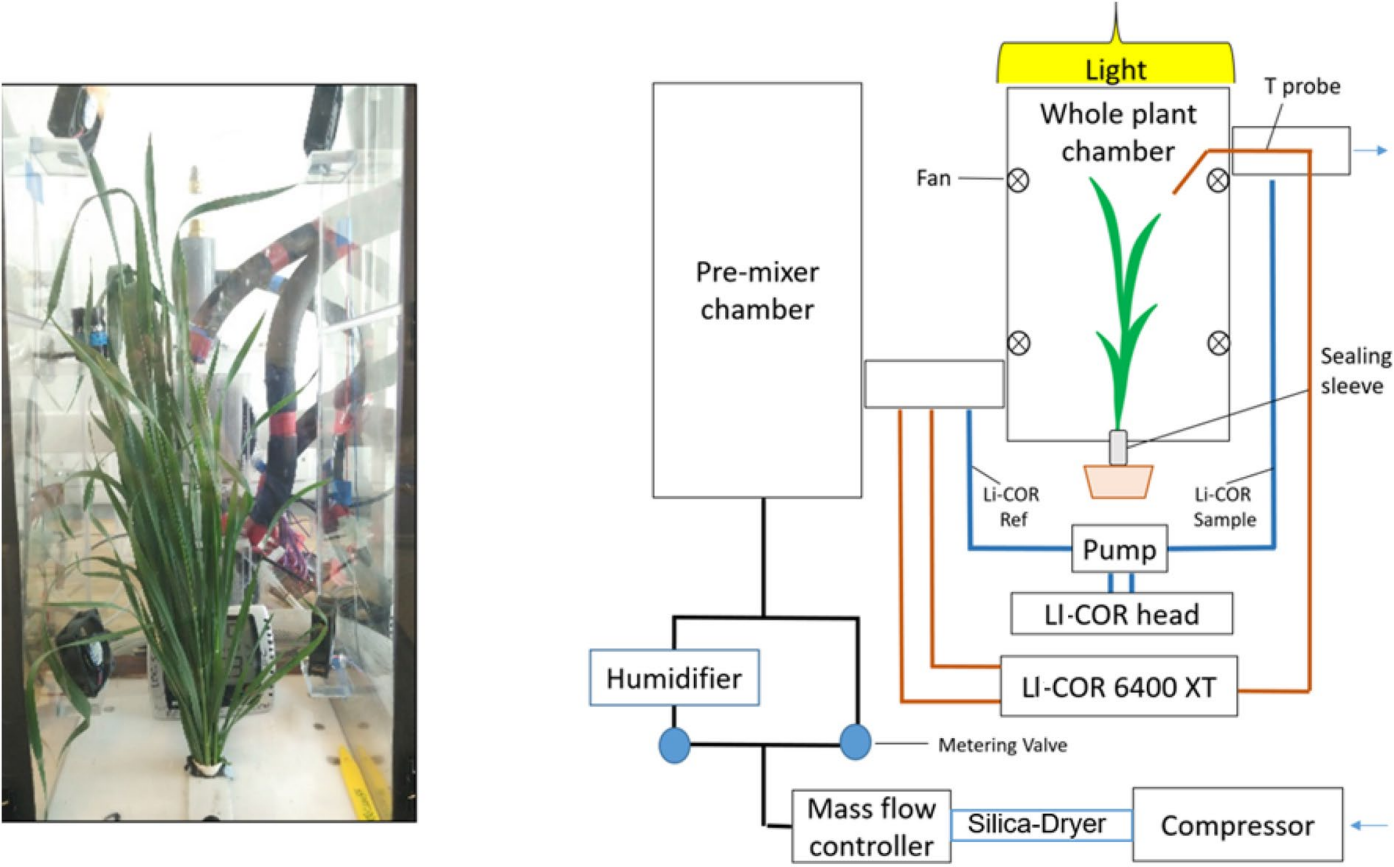
Picture (**a**) and schematic overview (**b**) of the whole plant gas-exchange system

Wheat (*Triticum aestivum*) and barley (*Hordeum vulgare*) were pre-germinated on moistened filter paper (Whatman #1) in petri dishes. The dishes were covered with foil and placed in dark conditions at room temperature (24 °C ± 5%) for 48 h. Once germinated, two seeds were placed at about 2.5 cm depth in rectangular 2 l pots (10.5 × 10.5 × 20 cm height) containing a commercial growing substrate (Petersfield Products, UK) with a slow-release fertilizer (Osmocote, Scotts UK Professional, UK). After the first true leaf emerged, one of the plants was removed from each pot to maintain one plant per pot. Twelve days after transplanting, the plants were supported in a sealing sleeve (Fig. 1, Additional file 1: Fig. S1). The plants grew for 6 weeks until reaching the phenological stage Zadoks 39–42. Plants were watered every 2–3 days to reach drained capacity, the maximum water content of the substrate, and were randomly allocated in the greenhouse and rotated weekly to assure homogeneity.

Plants were placed in a naturally lit greenhouse at Lancaster University (54.0104°N, 2.7877°W) with supplementary lighting (14 h per day), and controlled temperature (lights turn off if air temperature exceeds 30 °C). To maintain atmospheric VPD lower than 2.5 kPa throughout a diurnal cycle, a ten heads humidifier (Growell, UK) was placed in the greenhouse, to avoid developmental VPD priming of plants growing in different periods in the greenhouse.

Table 1 describes the different experiments done. The wheat cultivars (cv.) Krichauff and Drysdale were chosen because they showed contrasting Tr under increasing VPD [16]. The wheat cultivars cv. Cadenza, Gatsby, Mercato, Gladiator, Zebedee were chosen because they showed contrasting leaf photosynthesis (An) in a field experiment [36]. The barley ABA-deficient mutant *Az34* mutant (and its corresponding wild-type, WT) was chosen since it shows reduced capacity to produce ABA in response to water deficit, caused by a pleiotropic deficiency in the molybdenum cofactor that decreases aldehyde oxidase activity, which catalyses the ultimate step in the ABA biosynthesis pathway [40]. This mutant has higher Tr than the wild-type (WT) Steptoe in an early stage, both under control VPD and after increasing air temperature and, therefore, VPD [41].

**Table 1:** A detailed explanation of the experiments done with plants at Zadok’s stage 39–42. Spring experiments occurred between March and April, while summer experiments occurred between August and September

Experiment	Question	No of genotypes	No of reps	VPD	Approx. time per measurement	Period
Exp. 1	Is there genetic diversity in whole plant gas exchange at a single VPD?	7 (Wheat)	5–6	2.5 kPa	15 min	Spring 2017
Exp. 2	Does whole plant iWUE vary in response to VPD?	4 (Wheat and Barley)	2–3	0.75–4 kPa	3–4 h	Spring 2017
Exp. 3	Are results robust across experiments at different times of year?	4 (Wheat and Barley)	2–3	0.5–3.75 kPa	3–4 h	Summer 2017
Exp. 4	What is the mechanism behind *Az34* response to ABA spraying?	2 (Barley)	4–5	1.5 kPa	7 min; sampling	Spring 2018

### Whole plant gas exchange system

We re-designed the whole plant gas exchange system previously described [18]. With the new configuration and upgrades, the equipment can measure An and iWUE, in addition to Tr, under increasing VPD (Fig. 1). Hereafter, transpiration determined with this chamber is termed Tr_IRGA_ to avoid confusion with Tr obtained by gravimetric methods. The new system incorporates: (1) a powerful humidifier/dehumidifier system (Additional file 1: Fig. S5) that can more rapidly change chamber relative humidity (5 min compared to ~ 30 min required previously [18]) allowing higher VPDs (> 4 kPa) while maintaining temperatures below 30 °C; (2) a mass flow controller to tightly control the flow in the system by allowing a certain amount of pressure from the compressor while the previous version [18] pulled in air via a fan (3) multiple probes within the chamber to monitor environmental conditions including temperature, relative humidity and light, which were absent in [18]; (4) a LI- 6400XT (LI-COR, Lincoln, NE, USA) to simultaneously measure the gas exchange by logging the data measured using the various probes. The diagrams of the different parts are supplied in Additional file 1: Figs. S1–S4.

### IRGA and external probes

A LI-6400XT equipped with a 9964-053 Sample Cell Outlet Manifold Kit (LI-COR, Lincoln, NE, USA) to reduce the gas analyzer sample volume, was used to determine CO_2_ and H_2_O vapour concentrations. Using a LI-6400XT allowed external probes to be connected to the console to calculate An and Tr_IRGA_, using a protocol provided by LI-COR (LI-6400 Portable Photosynthesis System, Application Note 2) to communicate with the external probes as well as the IRGA. A temperature-humidity probe (Vaisala Humitter 50Y, Helsinki, Finland), a flow rate transducer (TSI 8450, Aliflow Instruments, USA) and a temperature probe (LI-COR, Lincoln, Nebraska, USA) were added.

The LI-6400XT head was connected to the chamber using vinyl flexible tubing (Swagelok, UK) and aluminium tube fittings and adapters (Swagelok, UK). Gas was driven through the LI-6400XT head using an external pump (model TD-4X2NA, Brailsford & CO, USA), which tightly controlled the flow of air. The flow rate achieved was checked every week. All tubing was covered with thermal insulation to stabilise dew point temperatures.

### The chamber

A chamber of total volume of 30 l (25 × 20 × 60 cm) was built from Perspex, with a nominal thickness of 3.5 mm. Light was supplied by two Son-T high-pressure sodium lamps (Philips, Netherlands) providing 450 µmol m^−2^ s^−1^ PPFD at the top of the plants. The light action spectrum that it is transmitted into the chamber was measured between 200 and 1100 nm (at 25 nm intervals) by placing the spectroradiometer (SR9910-V7, Macam Photometrics, Livingston, UK) inside the closed chamber (Additional file 1: Fig. S6).

To insert the plant into the chamber, one side consisted of a removable door (see Additional file 1: Fig. S3a) sealed with 1 cm wide neoprene sponge rubber and closed using eight metal clips. A sealable slot at the base of the chamber (see sealing sleeve description) isolated the root and shoot of the plant.

**Fig. 2:**
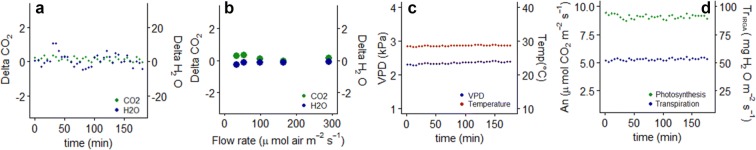
Reliability of the whole plant gas exchange system. Time course of differences (Delta) between air entering and exiting an empty chamber, for CO_2_ and H_2_O at a flow rate of 175 µmol air m^−2^ s^−1^ (**a**), and in response to flow rate (**b**); time courses for chamber vapour pressure deficit and air temperature with a plant in the system (**c**), and whole plant photosynthesis (An) and transpiration (Tr_IRGA_) of that plant (**d**)

The chamber is hermetically sealed and works under a slight overpressure. It is noteworthy that no leaks were detected in our system (Fig. 2a, b). Four fans (Ebmpapas 512Ft, Hungary) were placed inside the chamber (two fans on the top quarter and two fans on the bottom quarter of the chamber) to lower boundary layer resistance, with a combined capacity of 310 m^3^ h^−1^. Fan placement ensured homogenous airflow which was checked using smoke (data not shown). The equipment is operated in the laboratory, allowing the temperature to remain stable at 27.5 °C ± 5% when the fans are on (Fig. 2c). Temperature and relative humidity inside the chamber remains comparable (Additional file 1: Table S1).

### Sealing sleeve

A sealing sleeve, made of PVC (12 × 8 × 0.2 cm) (Additional file 1: Fig. S1) isolated the above and below-ground parts of the plant. In most cases, tiller development inside the sealing sleeve isolated the roots from the shoots, but to ensure gas tightness Sylgard Silicone elastomer (Dow Corning, UK) was applied inside the sealing sleeve 2 days prior to measurements. A neoprene sponge rubber ensured a tight fit of the plant into the chamber.

### The circuit

Air from outside the building was supplied to the chamber to assure a stable [CO_2_]. The [CO_2_] in this source changed less than 10 ppm during a typical day. To provide air under positive pressure, we used a compressor (OF1202-40MQ3, Junk Air, USA) with an extensive cooling system for temperature control. The compressed air was circulated through a 2 m pipe (1 cm internal ø) filled with silica gel to dehydrate it to approx. 5% RH. The silica gel was replenished after every 4–6 h of use. Thus conditioned, the air was supplied to the chamber at a stable rate of 30 l min^−1^ (with an error of 0.1%) via a mass flow controller (Alicat CMR 500 SLPM, Alicat Scientific, USA). This flow rate allows (1) an acceptable air renewal of one chamber volume per minute; (2) a reasonable [CO_2_] differential across the chamber (between − 18 to − 25 µmol CO_2_ m^−2^ s^−1^); (3) avoidance of high system pressure.

If necessary (at high VPDs or when plant leaf area exceeded 400 cm^2^), the flow rate was increased. A water bath (Fig. 1, Additional file 1: Fig. S2) containing an Ultrasonic humidifier with ten heads (Growell, UK) was developed to re-humidify the air (if needed). Manually operated low-pressure valves (Swagelok, UK) were used to control the amount of air passing through the water bath. The system established RH values in the range 5–75% by passing air through the water bath, and when higher RH was desired, the ultrasonic humidifier was connected. Although most of the tubing in the system has 0.4 cm internal ø, the tube that connects the humidifier system with the pre-mixer chamber has 1 cm internal ø to avoid condensation inside it.

To homogenize the air, it passes through a pre-mixer box (30 × 30 × 30 cm) (Fig. 1, Additional file 1: Fig. S4). Next, prior to entering the chamber, the air transits a PVC pipe (3 cm internal ø, 40 cm length) where flow rate, temperature and humidity probes and the reference line for LI-6400XT are assembled. The flow rate used for the gas exchange calculations is computed here. Typically, the conductance of this pipe averages 175 µmol air s^−1^ with an error of 5%. The pipe ends in the base of the chamber, circulating air upwards. Air exits the chamber via another PVC pipe where the sample line for the LI-6400XT is connected. A thermocouple (connected to the LI-6400XT) measures the temperature of a selected leaf from the top of the canopy.

### Data collection

At the beginning of each measurement sequence, the plants were acclimated for ~ 20 min to a VPD of 2.5 kPa, the maximum VPD experienced by plants in the greenhouse. Differences in [CO_2_] and [H_2_O] between air entering and exiting the chamber were measured and recorded using the LI-6400XT. Once the exchange of CO_2_ and H_2_O had been steady for more than 5 min (steady-state, Fig. 2d), values were logged every 20 s for 3–5 min, and a median value was established. Then, the relative humidity in the system was adjusted to inside the system were changed to achieve the next desired VPD level, usually requiring 15–30 min to reach a new steady-state. For VPD curves, VPD was gradually decreased to the minimum achievable in 0.5 kPa decrements. After that, VPD was increased in 0.5 kPa (or 0.75 kPa) increments to a maximum of 3.75 kPa during spring and above 4 kPa during summer experiments. Each plant was exposed to a minimum of 7 different VPDs. After measuring whole shoot gas exchange response to changing VPD, each plant was removed from the chamber to determine leaf area (LI-3100C Area Meter, Lincoln, NE, USA). Tr_IRGA_ did not significantly differ from gravimetrically determined Tr (see Additional file 1: Fig. S7).

To examine the effects of the *Az34* mutation in barley plants, leaf [ABA] was measured as previously described [12]. Frozen leaf tissues were freeze-dried and then powdered in a mortar. The ABA was extracted in distilled water (1:50, w/w) at 4 °C overnight in a shaker. ABA concentration was determined in aqueous extracts by a radioimmunoassay with the monoclonal antibody MAC252 as previously described [42]. The assay was conducted with two technical replicates per biological sample (Additional file 1: Table S2).

In some experiments, ABA was sprayed on the leaves to inhibit Tr. ABA was dissolved in ethanol to make a stock solution at 0.05 M, which was diluted to 10 µM in H_2_O prior to use. ABA was applied with a wetting agent Silwet (L-77, De Sangosse Ltd, Cambridge, UK) at 0.025%. We applied 10-15 ml per plant, depending on leaf area, using an atomizer (Perfume Pod, Amazon, UK). ABA-sprayed plants were used to measure whole plant gas exchange after 1 h (Additional file 1: Fig. S7).

Flag leaf gas exchange measurements were also made as part of these experiments spraying ABA over whole plants. The conditions in the LI-6400XT chamber were 1.5 kPa air VPD (to avoid stomatal limitations at high VPD), 500 µmol s^−1^ air flow, 400 ppm CO_2_, 25 °C leaf temperature (same as the in vitro Rubisco assay) and 460 µmol m^−2^ s^−1^ PPFD.

Flag leaf Rubisco in vitro activity was measured with a non-radioactive spectrophotometric assay with the modifications described by [43, 44]. The assay uses five enzymatic reactions to couple ribulose 1,5-bisphosphate (RuBP) carboxylation and 3-PGA formation to NADH oxidation. Rubisco activity is calculated based on RuBP consumption by monitoring the decrease in NADH concentration in the well, tracking the absorbance at 340 nm using UV-transparent 96-well plates in a microplate reader (SpectroStars, BMG Labtech, Germany) at 25 °C. Firstly, leaves were extracted as described by [36]. The Rubisco total activity (*Vt*) was assayed after incubating the extract for 5 min in the presence of CO_2_ and MgCl_2_, while the initial activity (*Vi*) was measured directly after extraction. The Rubisco activation state is the ratio *Vi*/*Vt*.

### Statistical analysis

One- or two-way ANOVA [45] was used to to test statistical significance of differences in means of each trait between genotypes or between genotypes and ABA treatments, respectively. Where significance of effects was observed (*P *< 0.05), multiple pairwise comparisons between treatments used the Tukey-b test.

To detect the Tr_IRGA_ breakpoint, the R package “segmented” [46] was used. When the results lacked biological meaning (resulting from statistical artefacts associated with exceeding the VPD operating boundaries of the chamber), or when the slope after the breakpoint was higher than the one before, a linear regression was used. Breakpoint calculations were made for each plant individually (Additional file 1: Tables S3–S5). Regression results were confirmed using the software Prism 7 (GraphPad Software Inc., San Diego, USA; Additional file 1: Table S6).

## Results

### Reliability of the whole plant system

Without a plant inside the chamber, delta H_2_O and delta CO_2_ were stable over time at a steady flow rate (Fig. 2a), and at different flow rates (Fig. 2b), indicating that leaks were absent or minimal. Rapid and large VPD changes (0.5–4 kPa) were possible in just a few min (Additional file 1: Fig. S5) while maintaining a temperature of 27.5 °C ± 5%, which is faster than in previously reported chambers [7, 18, 34, 35].

The whole plant system shows similar stability with a plant inside the chamber. Temperature and relative humidity were stable over time (Fig. 2c) because (1) the system was mounted in a temperature-controlled laboratory, and (2) the pipes were thermally insulated. Moreover, the water bath design (Additional file 1: Fig. S2) assured stability of VPD (Fig. 2c). With a wheat plant in the chamber, whole plant An and Tr_IRGA_ remained stable over time (Fig. 2d). Tr_IRGA_ measurements did not produce different results from paired gravimetric measurements (Additional file 1: Fig. S7).

### Whole plant gas exchange at a single VPD

**Fig. 3:**
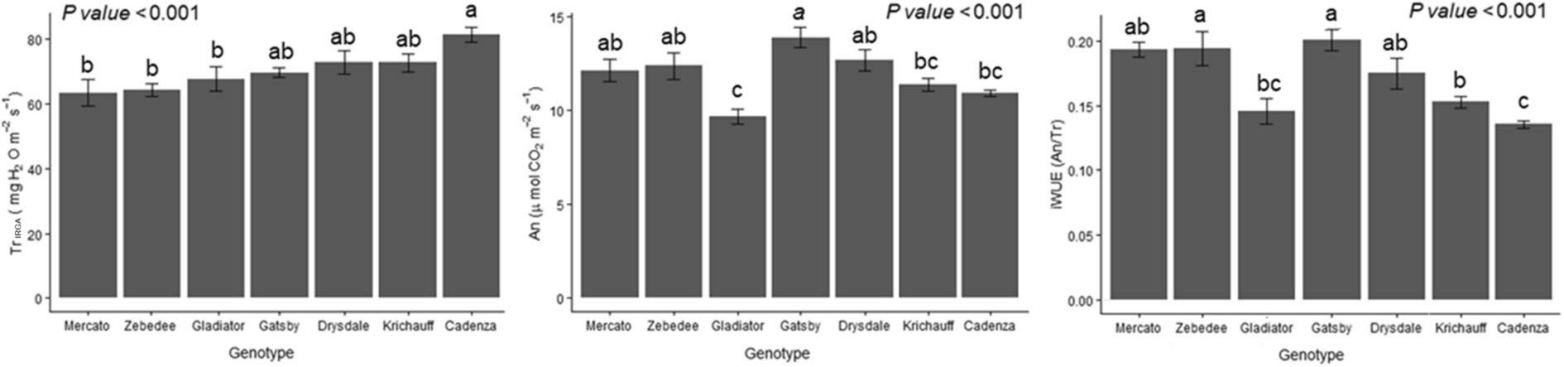
Whole plant transpiration (Tr_IRGA_), photosynthesis (An) and intrinsic water use efficiency (iWUE) in different wheat cultivars at a single VPD (2.5 kPa ± 6%). Bars are mean ± SE of 5–6 biological replicates, with different letters indicating significant (*P* < 0.05) differences according to a Tukey test

At a single VPD (2.5 kPa ± 6%) and constant temperature (27.5 °C ± 1%), different wheat cultivars showed significant differences in whole plant gas exchange (Fig. 3). Transpiration varied ca. 17%, with Cadenza having higher Tr_IRGA_ than Mercato, Zebedee and Gladiator, while Gatsby, Drysdale and Krichauff had intermediate values (Fig. 3a). Photosynthesis varied ca. 30%, with Gatsby having higher An than Gladiator, Krichauff and Cadenza, while Mercato, Zebedee and Drysdale had intermediate values (Fig. 3b). iWUE was more influenced by whole plant An than Tr_IRGA_ (Fig. 3c). Gatsby and Zebedee had higher iWUE than Cadenza, Gladiator and Krichauff, with Mercato and Drysdale having intermediate values. Since whole plant iWUE of Drysdale and Krichauff was similar, their gas exchange was studied under contrasting VPD levels.

### Effects of changing VPD on whole plant gas exchange

**Fig. 4:**
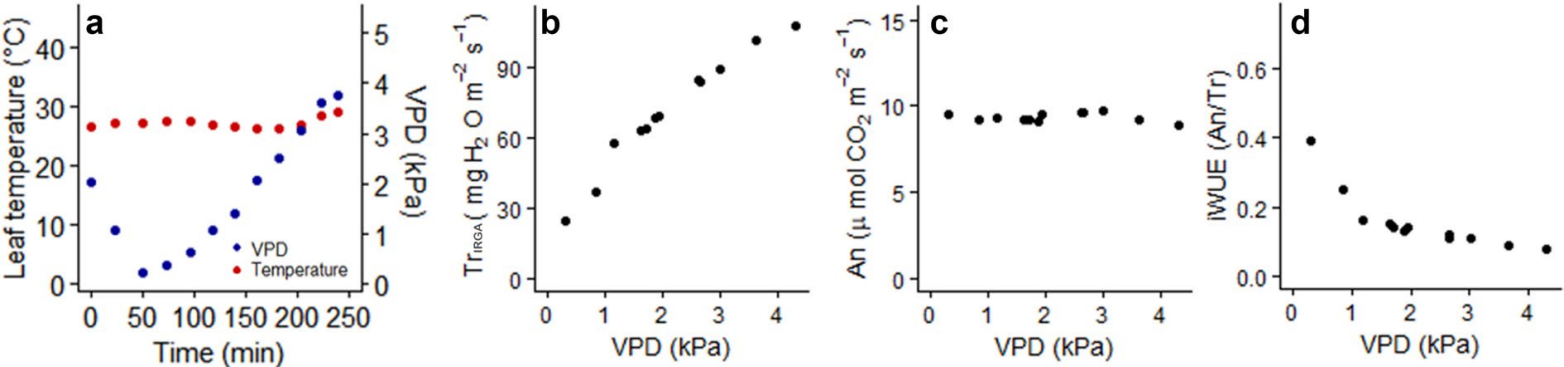
Whole plant gas exchange versus vapour pressure deficit (VPD) for a representative barley (cv. Steptoe) plant. **a** Chamber VPD and leaf temperature; **b** whole plant transpiration (Tr_IRGA_), **c** whole plant net photosynthesis (An), and **d** intrinsic water use efficiency (iWUE) versus VPD. Each point represents a median value for data collected at 20 s intervals over 3–5 min of steady state gas exchange, steady state at different VPD having been established every 20–30 min

A representative example of the data required to examine the presence of the Tr_IRGA_ breakpoint (BP) is shown in Fig. 4. Measurements commenced at 2.5 kPa, the maximum VPD experienced by plants in the greenhouse; then VPD was decreased to the minimum achievable in 0.5 kPa steps (Fig. 4a). After that, VPD was increased in 0.5–0.75 kPa steps to a maximum of 3.75 kPa. Air temperature inside the chamber remained stable during data collection (Fig. 4a). Following this protocol, plant gas exchange usually equilibrates within about 15–30 min because of the small (usually 0.5 kPa) VPD changes over time, and because, as grasses, *Triticum spp*. show relatively rapid stomatal movement due to their stomatal conformation [47]. Each VPD response curve took 3–4 h, and no pronounced hysteresis was detected when plants were exposed to ascending and descending series of VPDs (Additional file 1: Fig. S9). To avoid hydraulic limitations of transpiration that occur if the upper layers of the substrate dry out [48], water was added to the pot every hour during measurement until leaching was observed (since the pot could be irrigated without opening the chamber).

### Restriction of whole plant gas exchange under high vapour pressure deficit

**Fig. 5:**
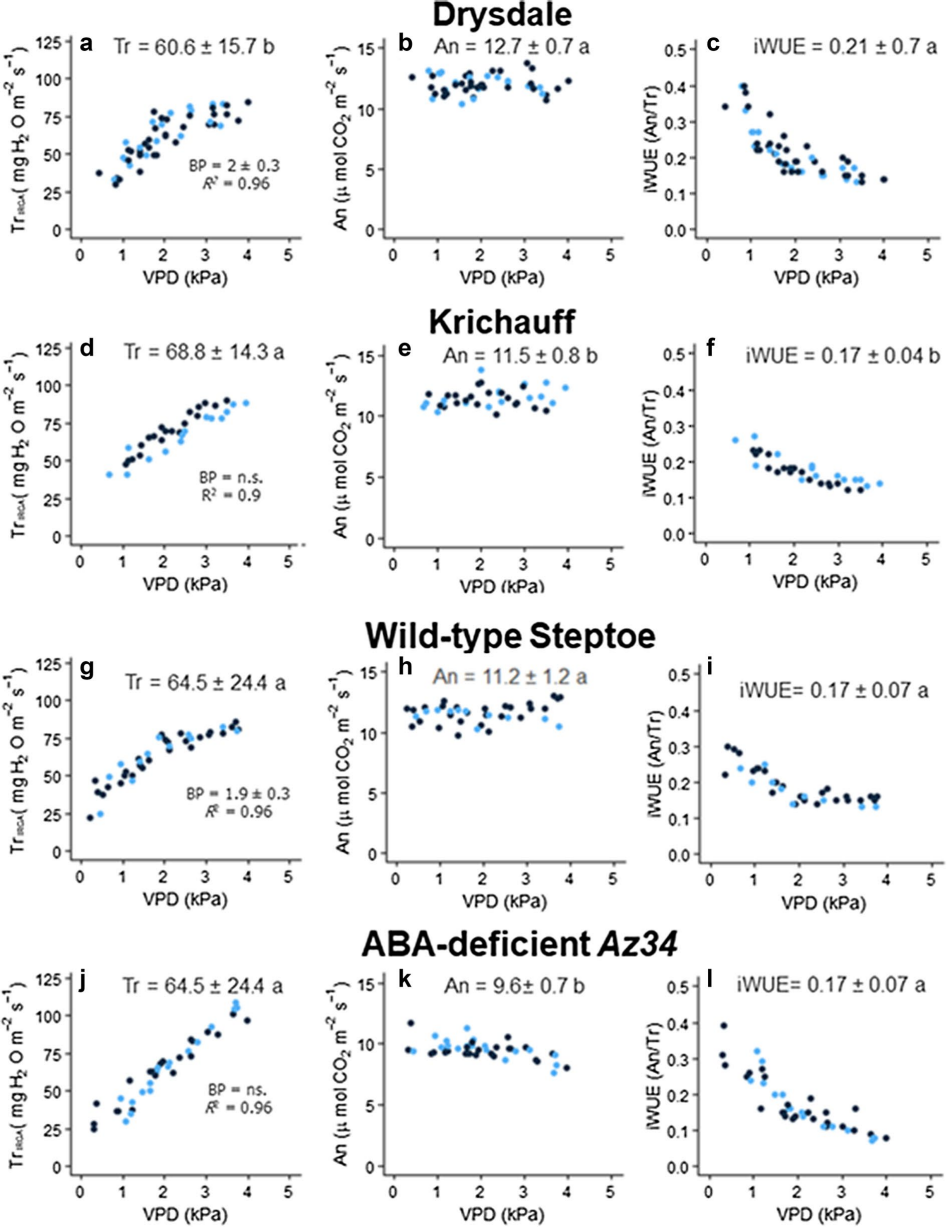
Whole plant transpiration (Tr_IRGA_), net photosynthesis (An) and intrinsic water use efficiency (iWUE) in the wheat cultivars Drysdale (**a**–**c**) and Krichauff (**d**–**f**), and the wild-type barley cultivar cv. Steptoe (**g**–**i**) and its ABA-deficient mutant *Az34* (**j**–**l**). Each point is from an individual plant, for 5–6 different plants per genotype. Dark blue dots represent plants measured in March–April 2017 while light blue dots represent plants measured in August–September 2017. Average values for each data set are indicated, with letters indicating significant (*P* < 0.05) differences between means when comparing either wheat or barley genotypes. Regression and break point analysis are available in Additional file 1: Tables S1–S3, with summary information included in each graph; *ns*, no significant difference

In the wheat cv. Drysdale, Tr_IRGA_ increased with increasing VPD and showed a BP at 2 ± 0.3 kPa (*R*^*2*^ = 0.96), while in cv. Krichauff, Tr_IRGA_ increased linearly with VPD (*R*^*2*^ = 0.91) (Fig. 5a, d; Additional file 1: Table S3), as previously described [16]. Across the entire range of VPDs, Tr_IRGA_ was significantly (*P *= 0.002) higher in cv. Krichauff than cv. Drysdale. When comparing Tr_IRGA_ below the Drysdale BP, both cultivars showed similar sensitivity of Tr_IRGA_ to VPD (same slope, *P* = 0.21). Beyond this BP, the slopes significantly differ (*P* = 0.003) with Tr_IRGA_ less sensitive to VPD in cv. Drysdale. Drysdale plants had a significantly (*P *= 0.003) higher An. Taken together, cv. Drysdale had a significantly (*P* = 0.005) higher iWUE than cv. Krichauff over the entire VPD range.

In wild-type (WT) barley, transpiration increased linearly with VPD up to 1.9 ± 0.3 kPa (*R*^*2*^ = 0.96), but VPDs above this threshold restricted transpiration (Fig. 5g, Additional file 1: Table S3). In contrast, transpiration of the ABA-deficient *Az34* barley mutant increased linearly and continuously with increasing VPD (*R*^*2*^ = 0.91) (Fig. 5j). Absolute Tr_IRGA_ of the *Az34* mutant was similar to WT over the entire VPD range. Before the BP at 1.9 kPa, the slope of the Tr_IRGA_ versus VPD response was similar (*P *= 0.55) between genotypes; beyond this BP, Tr_IRGA_ was more sensitive to VPD in the WT (*P *= 0.004). An was significantly lower in *Az34* than WT plants (*P* < 0.001), and decreased as VPD increased for the mutant only (Fig. 5h, k; Additional file 1: Table S4). *Az34* plants had a significantly (*P *= 0.002) lower An than WT both before (*P* < 0.001) and after (*P* < 0.001) the BP (1.9 kPa). In *Az34* plants, iWUE decreased exponentially as VPD increased, while in WT plants, iWUE decreased as VPD increased in the low range, but remained stable once VPDs exceeded the BP (Fig. 5i, l). Across the entire range of VPDs, iWUE did not significantly differ between genotypes. However, *Az34* had a significantly (*P* < 0.001) lower iWUE after the BP (1.9 kPa). Thus, the importance of ABA in determining iWUE of these genotypes varied according to the VPD.

### Differences in whole plant gas exchange in response to ABA in an ABA-deficient mutant

Before applying ABA and while at 2.5 kPa VPD, Tr_IRGA_ and An were 13% higher in WT than *Az34* plants, while iWUE did not significantly differ between genotypes (Table 2). Foliar ABA application reduced Tr_IRGA_ within 5 min in both the ABA-deficient mutant *Az34* and WT barley, with Tr_IRGA_ stabilising after 1 h (Additional file 1: Fig. S7). Whole plant Tr_IRGA_ decreased by 40 and 23% in *Az34* and WT plants respectively (Table 2); with the response almost significantly greater in *Az34* (*P* = 0.053 for genotype × ABA interaction). Interestingly, ABA application did not significantly affect An of WT plants, but decreased An by 30% in *Az34*. ABA treatment increased iWUE similarly in both genotypes (no significant genotype × ABA interaction). Taken together, whole plant gas exchange of the ABA-deficient mutant *Az34* was more responsive than WT plants to foliar ABA application.

**Table 2:** Whole plant transpiration (Tr_IRGA_, mg H_2_O m^−2^ s^−1^), photosynthesis (An, µmol CO_2_ m^−2^ s^−1^) and intrinsic water use efficiency (iWUE, µmol CO_2_ mg^−1^ H_2_O) before and after foliar application of ABA to the leaves of the wild-type (WT) barley genotype Steptoe and the ABA-deficient mutant *Az34*

	Whole plant Tr_IRGA_	Whole plant An	Whole plant iWUE
WT	75.3 ± 5.0	11.9 ± 0.9	0.16 ± 0.01
WT + ABA	62.4 ± 10.7	12.4 ± 1.7	0.20 ± 0.02
% change after ABA treatment	− 17	N.s.	+ 26
*Az34*	65.5 ± 3.8	10.3 ± 0.1	0.16 ± 0.01
*Az34* + ABA	39.4 ± 6.8	7.2 ± 1.4	0.18 ± 0.01
% change after ABA treatment	− 40	− 30	+ 18
Genotype	< 0.001	< 0.001	0.093
ABA treatment	< 0.001	0.025	< 0.001
Genotype * ABA treatment	0.053	0.005	0.093

The percentage change in response to ABA treatments is calculated when significant. Data are mean ± SE of 4 replicates. Results from two-way ANOVA (*P* values) are presented for Genotype, ABA treatment and their interaction

*N.s.* not significant

### Leaf-level measurements

To further investigate the mechanisms by which ABA limits photosynthesis, the flag leaves of *Az34* and WT plants were sprayed with ABA (Table 3). Stomatal conductance and leaf internal CO_2_ concentration (Ci) were 50 and 15% higher, respectively, in *Az34* than WT plants in the greenhouse prior to applying ABA. Following ABA application, both gs and Ci decreased, more severely in *Az34* plants as indicated by significant (*P* < 0.001 and *P* < 0.009) genotype × ABA interactions (Table 3). *Az34* had ca. 50% less total soluble protein (TSP) and Rubisco *Vt* than WT plants. *Az34* showed higher Rubisco activation state than WT prior to ABA application, with the opposite observed after ABA application as indicated by the significant (*P* < 0.001) genotype × ABA interaction (Table 3). ABA had no significant effect (*P* > 0.05) on TSP or Rubisco *Vt* for either genotype. However, while activation states were not affected by the ABA treatment in WT plants, *Az34* significantly reduced the activation state by ca. 25% (Table 3). Flag leaf ABA concentration of *Az34* was approximately half of the value of WT plants before spraying, and ABA application increased leaf [ABA] of both genotypes by sixfold to sevenfold (Additional file 1: Table S5). Taken together, stomatal and photosynthetic responses of *Az34* were more responsive to exogenous ABA spraying, despite similar proportional changes in foliar ABA accumulation.

**Table 3:** Flag leaf stomatal conductance (gs, mol m^−2^ s^−1^), internal [CO_2_] (Ci, µmol CO_2_ mol^−1^ air), total soluble proteins (TSP, g m^−2^), total Rubisco activity (*Vt*, µmol CO_2_ m^−2^ s^−1^) and Rubisco activation state (Act%, %) before and after foliar application of ABA to the leaves of the wild-type (WT) barley genotype Steptoe and the ABA-deficient mutant *Az34*

	Leaf gs	Leaf Ci	Leaf TSP	Leaf Rubisco *Vt*	Leaf Rubisco Act %
WT	0.26 ± 0.01	292 ± 5	7.44 ± 1.56	46.9 ± 12.1	65.9 ± 7.5
WT + ABA	0.14 ± 0.03	245 ± 29	8.10 ± 1.54	43.7 ± 10.4	66.4 ± 3.2
% change after ABA treatment	− 47	− 16	N.s.	N.s.	N.s.
*Az34*	0.39 ± 0.05	332 ± 9	3.57 ± 0.39	17.0 ± 1.4	76.0 ± 2.9
*Az34* + ABA	0.08 ± 0.02	234 ± 14	3.81 ± 0.4	19.8 ± 2.8	56.3 ± 3.8
% change after ABA treatment	− 79	− 30	N.s.	N.s.	− 26
Genotype	0.017	0.081	< 0.001	< 0.001	0.601
ABA treatment	< 0.001	< 0.001	0.839	0.960	< 0.001
Genotype * ABA treatment	< 0.001	0.009	0.200	0.450	< 0.001

The percentage change in response to ABA treatments is calculated when significant. Data are mean ± SE of 4 replicates. Results from two-way ANOVA (*P* values) are presented for Genotype, ABA treatment and their interaction

*N.s.* not significant

## Discussion

### The whole plant chamber can identify genetic diversity in gas exchange

A whole plant gas exchange chamber was adapted to study plant Tr_IRGA_, An and iWUE responses to changing VPD. The findings with wheat and barley genotypes support the idea that the chamber enables a robust assessment of these responses. A previous study demonstrated that some wheat genotypes restrict Tr at high VPD, such as cv. Drysdale [16], here we also show that this response significantly improves iWUE since photosynthesis is not limited above the BP. This reinforces the idea that iWUE can be improved by including the restricted transpiration trait at high VPD in those wheat genotypes that do not show it. In such genotypes, since An is not limited by VPD, this is an effective strategy to implement in breeding programs for drought-prone environments in elite plants [49, 50].

The whole plant gas exchange system was developed for phenotyping whole plant iWUE at different VPDs, and identified genetic differences. At a single VPD, genotypic differences in An correlated with single-leaf measurements done in field conditions in a previous experiment [36]. Nevertheless, at that specific VPD, a similar Tr_IRGA_ was found between Drysdale and Krichauff, in contrast with the results when comparing such genotypes under different VPD, reinforcing the importance of the VPD response curves in ranking Tr_IRGA_. It is important to note that plants were exposed to high VPD by maintaining air temperatures lower than 30 °C, which does not limit wheat photosynthesis [51, 52]. However, under natural conditions, high VPD and temperatures occur together, with inhibition of An by high VPD attributed to excessively high leaf temperatures [53]. Moreover, under non-steady state conditions, high VPD can constrain photosynthetic induction: the time required to reach the maximum An after the transition from low to high light [54]. Taken together, our results show that restricting Tr_IRGA_ at high VPDs at an optimal temperature range and under steady-state conditions does not affect carbon assimilation in commercial wheat and barley cultivars. It is essential to understand the physiological mechanisms regulating these responses.

### Determining the role of ABA in VPD responses

Previous measurements at whole plant level using gravimetric methods [8, 41] have implicated ABA in regulating cereal transpiration under varying evaporative demands. Similarly, transpiration of the ABA-deficient barley mutant *Az34* was unrestricted at high VPDs, but unexpectedly, An was limited (Fig. 4k; Additional file 1: Table S3). Single-leaf measurements were required to confirm the mechanistic response to the reduction of photosynthesis in ABA-deficient plants. Despite higher intercellular CO_2_ concentrations due to greater stomatal opening, *Az34* had a lower Rubisco activity (ca. 70% reduction compared to WT plants). Since *Az34* is nitrate-reductase deficient [40], plants are expected to be N limited with approximately half the total soluble protein content compared to WT plants (Table 3). Thus, the limited biomass of *Az34* not only results from its inability to control water loss under moderate-high VPD [41, 55], which induces leaf water deficit, but also from reduced Rubisco carboxylation that lowers photosynthesis.

To further demonstrate that dynamic whole plant responses can be detected with our system, ABA was sprayed on the leaves [12, 39]. Exogenous ABA application decreased Tr by ca. 25% in WT plants but even more so in *Az34* (by 40%), indicating greater stomatal sensitivity of the ABA-deficient mutant. These differences in whole plant transpiration sensitivity to ABA were confirmed in flag leaves (Table 3). Several ABA-deficient mutants in *Arabidopsis* (*aba2*-*11, nced3 nced 5, aba1*-*1, aba4*-*3, aao3*-*2, aba3*-*1)* and other species (*wilty* pea and *flacca* tomato) were described as hypersensitive to exogenous ABA application [35], attributed to a higher pre-treatment gs. Further work is required to investigate possible feedback regulation of genes for ABA sensitivity by ABA status in ABA-deficient mutants.

The mechanisms by which exogenous ABA limits photosynthesis remain under debate. While stomatal closure after ABA application decreases Ci even in ABA-deficient mutants ([39]; Table 3 here), the ABA molecule has been suggested to bind to the Rubisco active site blocking Rubisco activity [56]. While foliar ABA spraying did not affect photosynthesis of WT plants (Table 2), An was decreased by 30% in *Az34*, as in a previous comparison of WT and ABA-deficient tomatoes (*flacca* mutant) grown under non-saturating light (Bradford et al. 1983). ABA application decreased Rubisco activation state of *Az34* flag leaves but not WT leaves (Table 2). Similar to in vitro ABA experiments [56], Rubisco activation of such plants might be disrupted by the ABA molecule. Alternatively, *Az34* may have higher CO_2_ availability under standard conditions in the greenhouse (before ABA application). The larger decreases in gs and Ci observed in *Az34* after ABA application may deactivate Rubisco because of the limited CO_2_ availability, thereby decreasing photosynthesis. Whether such limitations occur because stomatal and mesophyll conductance are co-ordinated [57], or due to a mechanistic constraint of ABA on Rubisco activity, is still unknown. In either case, the lower Rubisco activity of *Az34* makes its photosynthesis more vulnerable to environmental constraints, such as high VPD, than WT plants.

## Conclusions

Our chamber was designed, built and operated to evaluate whole plant An, Tr_IRGA_ and iWUE under increasing evaporative demand in small-grain cereals. This instrumentation is sufficiently precise to detect genetic differences in plant responses. In wild-type genotypes, photosynthesis was not restricted by VPD “per se”, even though some genotypes restricted Tr_IRGA_ under high VPD, which is of direct interest to plant breeders seeking to increase iWUE. Furthermore, ABA-deficient barley responded more sensitively to exogenous ABA application, with greater transpirational restriction and decreased Rubisco activation state. Photosynthesis of ABA-deficient barley plants was also limited at high VPD, likely due to reduced Rubisco activity.

## Additional file

**Additional file 1.** Supplemental tables and figures.
