# Supplementary material for: Whole plant chamber to examine sensitivity of cereal gas exchange to changes in evaporative demand
Source: Plant Methods. 2018 Nov 1;14:97. doi: 10.1186/s13007-018-0357-9 (PMC6211548; doi:10.1186/s13007-018-0357-9)
Supplement: Supplementary file 1 — Additional file 1. Supplemental tables and figures. [file 13007_2018_357_MOESM1_ESM.docx]

**Supplemental table 1**. Environmental conditions measured within the whole plant gas exchange chamber with 4 independent probes, those of the chamber (Vaisala Humitter 50Y, Helsinki, Finland) and 3 external probes (Omega EL-USB-2, Manchester, UK) measured during 1 h. All probes were covered with foil to avoid heating (Final table will include a comparison of all plants). Values with the same letters within the same column are not significantly (P < 0.05) different.

|  | Temperature (°C) | Relative humidity (%) |
| --- | --- | --- |
| Measured by the probes of the chamber (see Supplemental Figure 3) | 27.43 ± 0.12 a | 55.8 ± 1.0 a |
| Measured at 10 cm above the chamber floor | 27.78 ± 0.24 a | 54.7 ± 1.1 a |
| Measured at 30 cm above the chamber floor | 27.50 ± 0.01 a | 56.8 ± 1.5 a |
| Measured at 50 cm above the chamber floor | 27.42 ± 0.18 a | 56.8 ± 1.0 a |

**Supplemental Table 2.** Flag leaf ABA concentration (ng g^-1^ dry weight) in the wild-type (WT) barley genotype Steptoe and the ABA-deficient mutant *Az34* either sprayed (or not) with ABA. Data are means ± SE of 4-6 replicate plants. Two-way ANOVA (*P-value*) at base of Table indicates significant effects of genotype, ABA treatment and interaction, with *P*-values within Table for pairwise comparison of effects of ABA spraying.

|  | [ABA] |
| --- | --- |
| WT (Steptoe) | 514 ± 24 |
| WT (Steptoe) + ABA | 3756 ± 1383 |
| *Az34* (ABA-deficient) | 209 ± 69 |
| *Az34* (ABA-deficient) + ABA | 1296 ± 406 |
| Genotype | <0.001 |
| ABA treatment | <0.001<0.001 |
| Genotype * ABA treatment | <0.001<0.001 |

**Supplemental Table 3**. Statists for whole plant transpiration rates measured our system (mg H_2_O m^-2^ s^-1^) in wheat and barley genotypes. Results of one-way ANOVA comparing different genotypes (*P* values) are presented.

| **Whole plant transpiration _IRGA_- individual plants** | | | | | | | |
| --- | --- | --- | --- | --- | --- | --- | --- |
| Genotype | Plant | BP | Slope 1 | Slope 2 | R^2^ | Linear model | R^2^ |
| Drysdale | Plant1 | 2.5 | 23.1 | 5.2 | 0.97 | - | - |
| Drysdale | Plant2 | 2.3 | 15.8 | 7..7 | 0.99 | - | - |
| Drysdale | Plant3 | 1.8 | 36.0 | 7.8 | 0.94 | - | - |
| Drysdale | Plant4 | 1.9 | 33.0 | 12.5 | 0.99 | - | - |
| Drysdale | Plant5 | 1.9 | 21.9 | 7.9 | 0.93 | - | - |
| Drysdale | Plant6 | 2.0 | 27.4 | 5.9 | 0.94 | - | - |
| Krichauff | Plant1 | - | - | - | - | 12.7 | 0.92 |
| Krichauff | Plant2 | 1.6 | 34.1 | 13.1 | 0.99 | 16.2 | 0.94 |
| Krichauff | Plant3 | 1.1 | 97.2 | 14.4 | 0.95 | 19.8 | 0.78 |
| Krichauff | Plant4 | 2.2 | 20.7 | 22.6 | 0.99 | 21.6 | 0.99 |
| Krichauff | Plant5 | - | - | - | - | 23.6 | 0.87 |
|  |  |  |  |  |  |  |  |
| WT | Plant1 | 1.7 | 28.7 | 5.6 | 0.96 | - | - |
| WT | Plant2 | 2.4 | 17.7 | 5.9 | 0.89 | - | - |
| WT | Plant3 | 1.8 | 20.9 | 9.4 | 0.99 | - | - |
| WT | Plant4 | 1.7 | 34.7 | 6.2 | 0.99 | - | - |
| WT | Plant5 | 1.9 | 19.3 | 5.6 | 0.97 | - | - |
| *Az34* | Plant1 | 1.0 | 36.7 | 19.2 | 0.98 |  | - |
| *Az34* | Plant2 | - | - | - | - | 10.4 | 0.93 |
| *Az34* | Plant3 | 1.8 | 37.2 | 25.1 | 1.0 | 29.6 | 0.98 |
| *Az34* | Plant4 | - | - | - | - | 22.1 | 0.99 |
| *Az34* | Plant5 | 2.2 | 12.9 | 17.8 | 0.97 | 15.4 | 0.97 |
|  |  |  |  |  |  |  |  |
| **Sensitivity of whole plant transpiration *versus* VPD compared before and after the transpiration BP** | | | | | | | |
| Genotype | | BP | R^2^ | Slope Tr < BP | *P-value Slope Tr < BP* | Slope Tr > BP | *P-value Slope Tr > BP* |
| Drysdale | | 2.0 ± 0.3 | 0.96 | 26.2 ± 7.4 | 0.083 | 7.8 ± 2.8 | <0.001 |
| Krichauff | | n.s. | 0.91 | 22.3 ± 7.7 |  | 16.4 ± 4.6 |  |
|  |  |  |  |  |  |  |  |
| WT | | 1.9 ± 0.3 | 0.96 | 24.2 ± 7.2 | 0.553 | 6.5 ± 1.6 | 0.005 |
| *Az34* | | - | 0.97 | 19.3 ± 7.2 |  | 19.3± 7.2 |  |
|  |  |  |  |  |  |  |  |
| **Whole plant transpiration - before and after the transpiration BP comparison** | | | | | | | |
| Genotype | | Tr | *P-value* | Tr, Tr < BP | *P-value < BP* | Tr, Tr > BP | *P-value > BP* |
| Drysdale | | 60.6 ± 15.7 | 0.020 | 50.8 ± 13.0 | 0.262 | 73.6 ± 8.00 | 0.246 |
| Krichauff | | 68.79 ± 14.28 |  | 55.4 ± 9.8 |  | 77.0 ± 9.6 |  |
|  |  |  |  |  |  |  |  |
| WT | | 67.2 ± 19.8 | 0.348 | 51.3 ± 14.1 | 0.553 | 76.7 ± 5.0 | 0.037 |
| *Az34* | | 63.2 ± 22.7 |  | 48.7 ± 14.2 |  | 84.0 ± 13.9 |  |

| **Supplemental Table 4**. Statist for whole plant photosynthesis (µmol CO_2_ m^-2^ s^-1^) in wheat and barley genotypes. Results of one-way ANOVA comparing different genotypes (*P* values) are presented).  **Whole plant photosynthesis - individual plants** | | | | | | | |
| --- | --- | --- | --- | --- | --- | --- | --- |
| Genotype | Plant | BP for A | Slope 1 | Slope 2 | R^2^ | Linear model | R^2^ |
| Drysdale | Plant1 | 3.5 | 0.6 | -7.1 | 0.05 | 0.2 | 0.05 |
| Drysdale | Plant2 | 3.5 | -0.4 | 2.1 | 0.27 | -0.1 | 0.48 |
| Drysdale | Plant3 | 3.0 | 0.3 | -0.1 | 0.75 | - | - |
| Drysdale | Plant4 | 2.6 | 0.6 | -1.0 | 0.55 | - | - |
| Drysdale | Plant5 | 2.5 | -1.3 | 3.1 | 0.88 | - | - |
| Drysdale | Plant6 | 3.1 | 0.3 | -3.1 | 0.65 | - | - |
| Krichauff | Plant1 | 2.3 | -0.1 | 0.0 | 0.97 | - | - |
| Krichauff | Plant2 | 2.1 | 1.8 | -1.7 | 0.78 | - | - |
| Krichauff | Plant3 | 2.0 | 1.4 | -0.2 | 0.46 | - | - |
| Krichauff | Plant4 | 2.5 | -1.3 | 3.1 | 0.88 | - | - |
| Krichauff | Plant5 | 3.1 | 0.3 | -3.1 | 0.65 | - | - |
|  |  |  |  |  |  |  |  |
| WT | Plant1 | 2.2 | -0.2 | 0.7 | 0.68 | - | - |
| WT | Plant2 | 2.6 | 0.4 | 1.3 | 0.83 | - | - |
| WT | Plant3 | 1.8 | -0.7 | 1.2 | 0.53 | 0.4 | 0.32 |
| WT | Plant4 | 1.5 | 0.6 | -0.6 | 1.00 | - | - |
| WT | Plant5 | 1.9 | -0.9 | 0.2 | 0.54 | -0.3 | 0.27 |
| *Az34* | Plant1 | - | - | - | 0.51 | -1 | 0.78 |
| *Az34* | Plant2 | 1.3 | 0.2 | -0.4 | 1.00 | - | - |
| *Az34* | Plant3 | 1.2 | 0.7 | -0.3 | 0.70 | - | - |
| *Az34* | Plant4 | - | -1.3 | -1.3 | 0.83 | -1.3 | 0.83 |
| *Az34* | Plant5 | - | -0.9 | -0.9 | 0.75 | -0.9 | 0.75 |
|  |  |  |  |  |  |  |  |
| **Sensitivity of whole plant photosynthesis *versus* VPD compared before and after the transpiration BP** | | | | | | | |
| Genotype | | BP | R^2^ | Slope Tr < BP | *P-value Slope Tr < BP* | Slope Tr > BP | *P-value Slope Tr > BP* |
| Drysdale | | 3.0 ± 0.4 | 0.52 | .-1.08 ± 3.69 | 0.519 | .-1.18 ± 4.12 | 0.748 |
| Krichauff | | 2.4 ± 0.4 | 0.8 | 0.42 ± 1.24 |  | .-0.39 ± 2.33 |  |
|  |  |  |  |  |  |  |  |
| WT | | 2.0 ± 0.4 | 0.7 | .-0.15 ± 0.67 | 0.87 | 0.7 ± 0.2 | 0.014 |
| *Az34* | | - | 0.76 | .-0.25 ± 0.84 |  | .-1.0 ± 0.2 |  |
|  |  |  |  |  |  |  |  |
| **Whole plant photosynthesis compared before and after the transpiration BP** | | | | | | | |
| Genotype | | A | *P-value* | A , Tr< BP | *P-value < BP* | A, Tr > BP | *P-value > BP* |
| Drysdale | | 12.0 ± 0.7 | 0.003 | 11.9 ± 0.8 | 0.59 | 12.2 ± 0.7 | 0.086 |
| Krichauff | | 11.51 ± 0.79 |  | 11.3 ± 0.5 |  | 11.7 ± 0.9 |  |
|  |  |  |  |  |  |  |  |
| WT | | 11.2 ± 1.2 | <0.001 | 11.4 ± 0.7 | <0.001 | 11.7 ± 0.8 | <0.001 |
| *Az34* | | 9.5 ± 0.7 |  | 9.8 ± 0.7 |  | 9.2 ± 0.7 |  |

**Supplemental Table 5**. Statist for whole plant intrinsic water use efficiency (iWUE; µmol CO_2_ mg^-1^ H_2_O) in wheat and barley genotypes. Results of one-way ANOVA comparing different genotypes (*P* values) are presented).

| **Whole plant instantaneous water use efficiency - individual plants** | | | | | | | | |
| --- | --- | --- | --- | --- | --- | --- | --- | --- |
| Genotype | Plant | BP for iWUE | Slope 1 | Slope 2 | | R^2^ | Linear model | R^2^ |
| Drysdale | Plant1 | 2.0 | -0.2 | 0.0 | | 0.97 | - | - |
| Drysdale | Plant2 | 1.5 | -0.1 | 0.0 | | 0.9 | - | - |
| Drysdale | Plant3 | 1.1 | -0.6 | 0.0 | | 0.98 | - | - |
| Drysdale | Plant4 | 1.3 | -0.3 | 0.0 | | 0.99 | - | - |
| Drysdale | Plant5 | 1.4 | -0.2 | 0.0 | | 0.96 | - | - |
| Drysdale | Plant6 | 2.1 | -0.1 | 0.0 | | 0.91 | - | - |
| Krichauff | Plant1 | 1.1 | -2.4 | 0.0 | | 1.00 | - | - |
| Krichauff | Plant2 | 1.1 | -2.4 | 0.0 | | 1.00 | - | - |
| Krichauff | Plant3 | 2.3 | -0.1 | 0.0 | | 0.97 | - | - |
| Krichauff | Plant4 | 2.1 | -0.1 | 0.0 | | 1.00 | - | - |
| Krichauff | Plant5 | 2.3 | -0.1 | 0.0 | | 0.97 | - | - |
|  |  |  |  |  | |  |  |  |
| WT | Plant1 | 0.4 | -1.5 | 0.0 | | 0.96 | - | - |
| WT | Plant2 | 2.4 | 0.0 | 0.0 | | 0.82 | - | - |
| WT | Plant3 | 1.6 | -0.1 | 0.0 | | 0.92 | - | - |
| WT | Plant4 | 1.5 | -0.3 | 0.0 | | 1.00 | - | - |
| WT | Plant5 | 1.9 | -0.1 | 0.0 | | 0.93 | - | - |
| *Az34* | Plant1 | - | - | - | | - | -0.3 | 0.9 |
| *Az34* | Plant2 | 2.3 | -0.1 | 0.0 | | 0.98 | - | - |
| *Az34* | Plant3 | 1.3 | -0.5 | -0.1 | | 0.97 | - | - |
| *Az34* | Plant4 | 3.5 | -0.1 | 0.2 | | 0.98 | - | - |
| *Az34* | Plant5 | - | - | - | | - | -0.1 | 0.89 |
|  |  |  |  |  | |  |  |  |
| **Whole plant instantaneous water use efficiency compared before and after the transpiration BP** | | | | | | | | |
| Genotype | | iWUE | *P-value* | iWUE, Tr< BP | | *P-value < BP* | iWUE, Tr > BP | *P-value > BP* |
| Drysdale | | 0.21 ± 0.7 | 0.005 | 0.25 ± 0.07 | | 0.052 | 0.17 ± 0.02 | 0.031 |
| Krichauff | | 0.17 ± 0.04 |  | 0.21 ± 0.03 | |  | 0.15 ± 0.03 |  |
|  |  |  |  |  | |  |  |  |
| WT | | 0.17 ± 0.06 | 0.497 | 0.22 ± 0.07 | | 0.615 | 0.15 ± 0.01 | <0.001 |
| *Az34* | | 0.18 ± 0.05 |  | 0.23 ± 0.07 | |  | 0.12 ± 0.03 |  |
|  | | | | |  |  |  |  |

**Supplemental table 6**. Comparing slope and break points (BP) fitted by non-linear regression between transpiration rates using our system and VPD obtained using the segmented R package and GraphPad Prism 7. (Final table will include a comparison of all plants)

|  |  |  | **Slope 1** | **Slope 2** | **BP** | **R^2^** |
| --- | --- | --- | --- | --- | --- | --- |
| Drysdale | Plant1 | Segmented | 23.1 | 5.2 | 2.5 | 0.97 |
|  |  | Prism 7 | 23.2 | 5.2 | 2.5 | 0.97 |
| Drysdale | Plant2 | Segmented | 15.8 | 7.7 | 2.3 | 0.99 |
|  |  | Prism 7 | 15.8 | 7.8 | 2.3 | 0.99 |
| Drysdale | Plant3 | Segmented | 36 | 7.8 | 1.8 | 0.94 |
|  |  | Prism 7 | 36.1 | 7.8 | 1.8 | 0.94 |
| Drysdale | Plant4 | Segmented | 33 | 12.5 | 1.9 | 0.99 |
|  |  | Prism 7 | 33 | 12.4 | 1.9 | 0.99 |
| Drysdale | Plant5 | Segmented | 21.9 | 7.9 | 1.9 | 0.93 |
|  |  | Prism 7 | 21.9 | 7.9 | 1.9 | 0.93 |
| Drysdale | Plant6 | Segmented | 27.4 | 5.9 | 2 | 0.94 |
|  |  | Prism 7 | 27.5 | 6 | 2.01 | 0.94 |
| Krichauff | Plant1 | Segmented | 12.7 | 12.7 | linear | 0.92 |
|  |  | Prism 7 | 30.5 | 20.9 | 1.1 | 0.74 |
| Krichauff | Plant2 | Segmented | 34.1 | 13.1 | 1.6 | 0.99 |
|  |  | Prism 7 | 37.1 | 12.1 | Ambiguous (~1.6) | 0.99 |
| Krichauff | Plant3 | Segmented | 97.2 | 14.4 | 1.1 | 0.95 |
|  |  | Prism 7 | 84.5 | 14.9 | Ambiguous (~1.0) | 0.96 |
| Krichauff | Plant4 | Segmented | 20.7 | 22.6 | 2.2 | 0.99 |
|  |  | Prism 7 | 20.6 | 25.5 | 2.7 | 0.99 |
| Krichauff | Plant5 | Segmented | 23.6 | 23.6 | linear | 0.99 |
|  |  | Prism 7 | 22.6 | 23.9 | Ambiguous (~2.2) | 0.97 |
| *WT* | Plant1 | Segmented | 28.7 | 5.6 | 1.7 | 0.96 |
|  |  | Prism 7 | 28.7 | 5.6 | 28.6 | 0.97 |
| *WT* | Plant2 | Segmented | 17.7 | 5.9 | 2.4 | 0.89 |
|  |  | Prism 7 | 17.7 | 5.9 | 2.4 | 0.89 |
| *WT* | Plant3 | Segmented | 20.9 | 9.4 | 1.8 | 0.99 |
|  |  | Prism 7 | 20.9 | 0.3 | 1.8 | 0.99 |
| *WT* | Plant4 | Segmented | 34.7 | 6.2 | 1.7 | 0.99 |
|  |  | Prism 7 | 34.7 | 6.2 | 1.7 | 0.99 |
| *WT* | Plant5 | Segmented | 19.3 | 5.6 | 1.9 | 0.97 |
|  |  | Prism 7 | 19.2 | 5.7 | 1.9 | 0.97 |
| *Az34* | Plant1 | Segmented | 36.7 | 19.2 | 1 | 0.98 |
|  |  | Prism 7 | 94.3 | 18.9 | Ambiguous (~1.27) | 0.98 |
| *Az34* | Plant2 | Segmented | 10.4 | 10.4 | linear | 0.93 |
|  |  | Prism 7 | 47.3 | 11.9 | Ambiguous (~1.0) | 0.87 |
| *Az34* | Plant3 | Segmented | 37.2 | 25.1 | 1.8 | 1 |
|  |  | Prism 7 | 37.1 | 24.3 | 1.81 | 0.98 |
| *Az34* | Plant4 | Segmented | 22.1 | 22.1 | linear | 0.99 |
|  |  | Prism 7 | 30.6 | 16.2 | 1 | 0.96 |
| *Az34* | Plant5 | Segmented | 12.9 | 17.8 | 2.2 | 0.97 |
|  |  | Prism 7 | 12.4 | 17.8 | 2.1 | 0.98 |

**
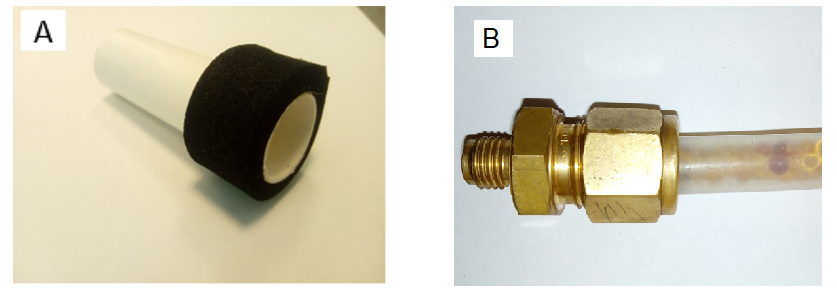
**

**Supplemental Fig. 1** The sealing sleeve (A) and silica gel inside the drying pipe (B).

**
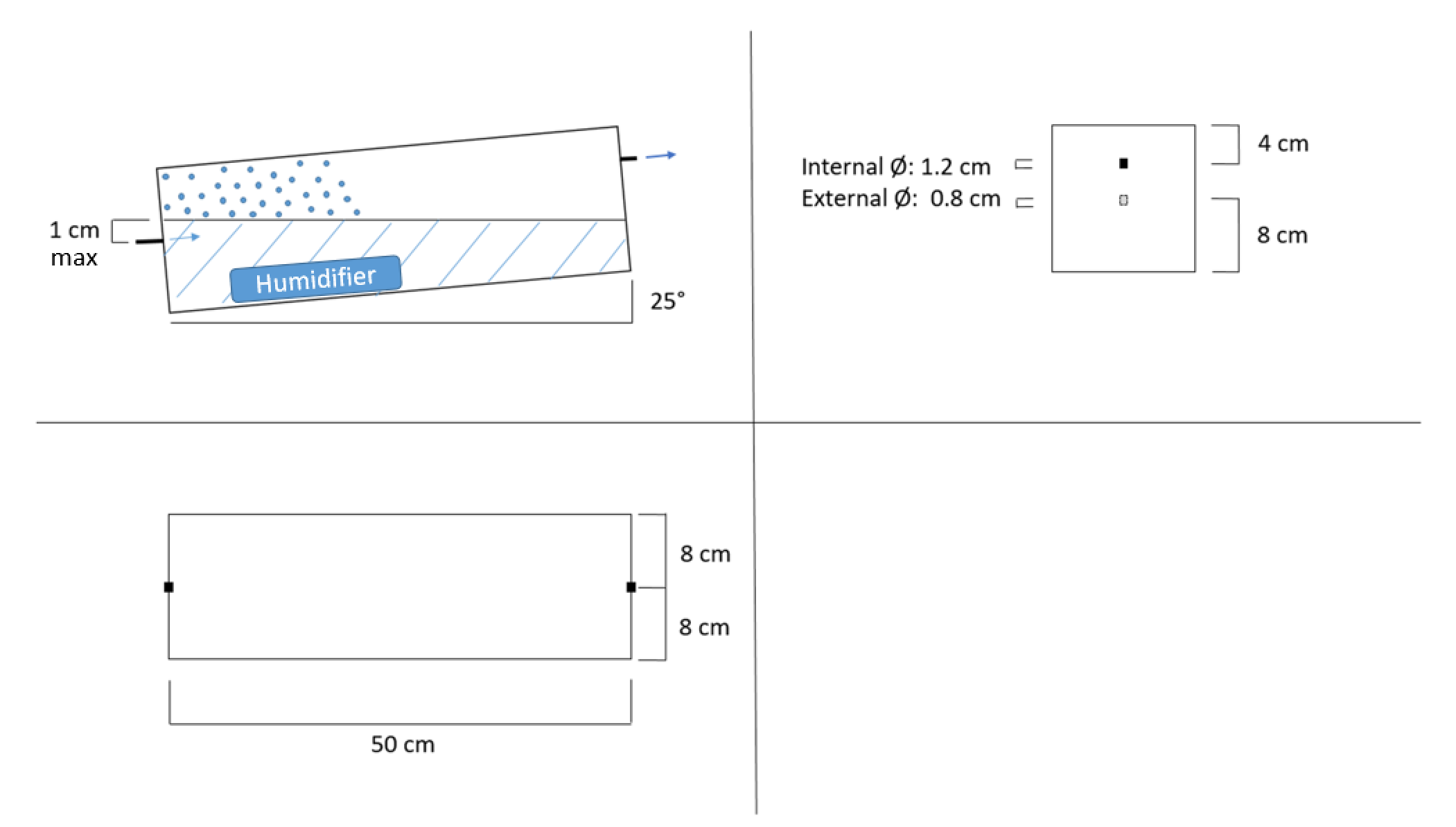
**

**Supplemental Fig. 2**. Plane of humidifier system in the whole plant gas exchange system: (A) side view, showing inclination of humidifier; (B) front view, with dimensions; (C) top view with dimensions and points of access.

**
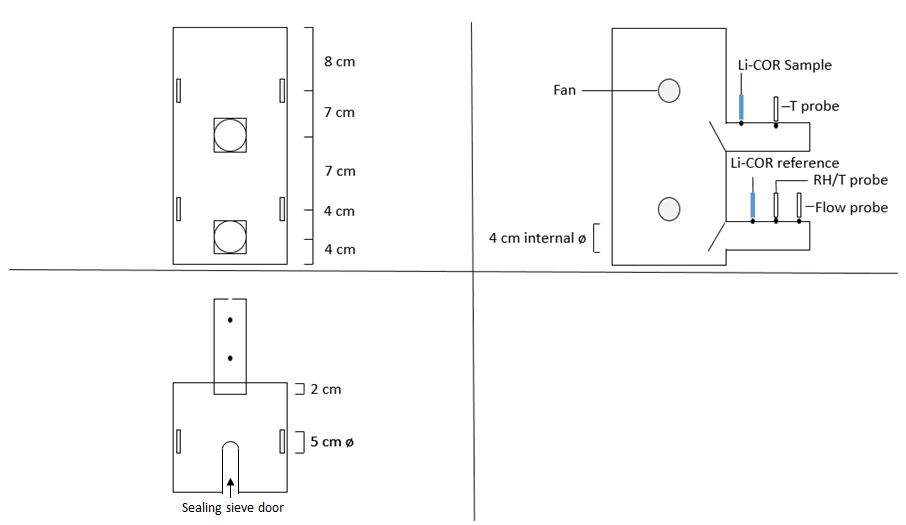
**

**Supplemental Fig. 3**. Plane of the chamber in the whole plant gas exchange system: (A) side view, distances between the fans; (B) front view, with connection of the different proves; (C) top view with dimensions and plant access across sealing sieve door.


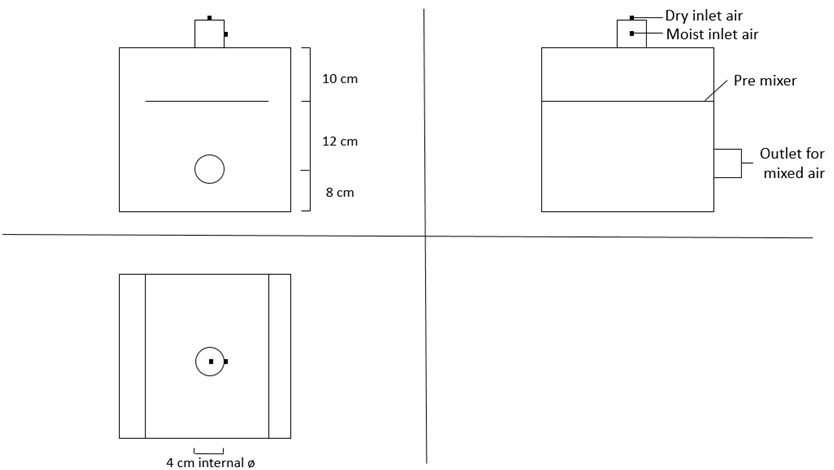


**Supplemental Fig. 4**. Plane of the pre-mixer chamber in the whole plant gas exchange system: (A) side view, distances between entrance and exit tubing; (B) front view, connection description; (C) top view with internal diameter of the tubbing.


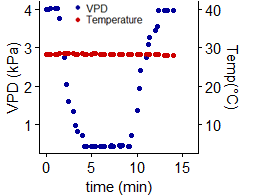


**Supplemental Fig. 5**. Capacity of the system to rapidly change VPD: VPD and air temperature (Temp) with a plant in the system.

**
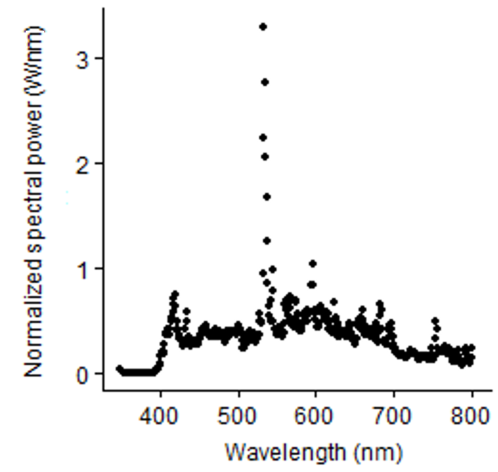
**

**Supplemental Fig. 6** Spectral distribution of energy from photosynthetically active radiation inside the chamber.


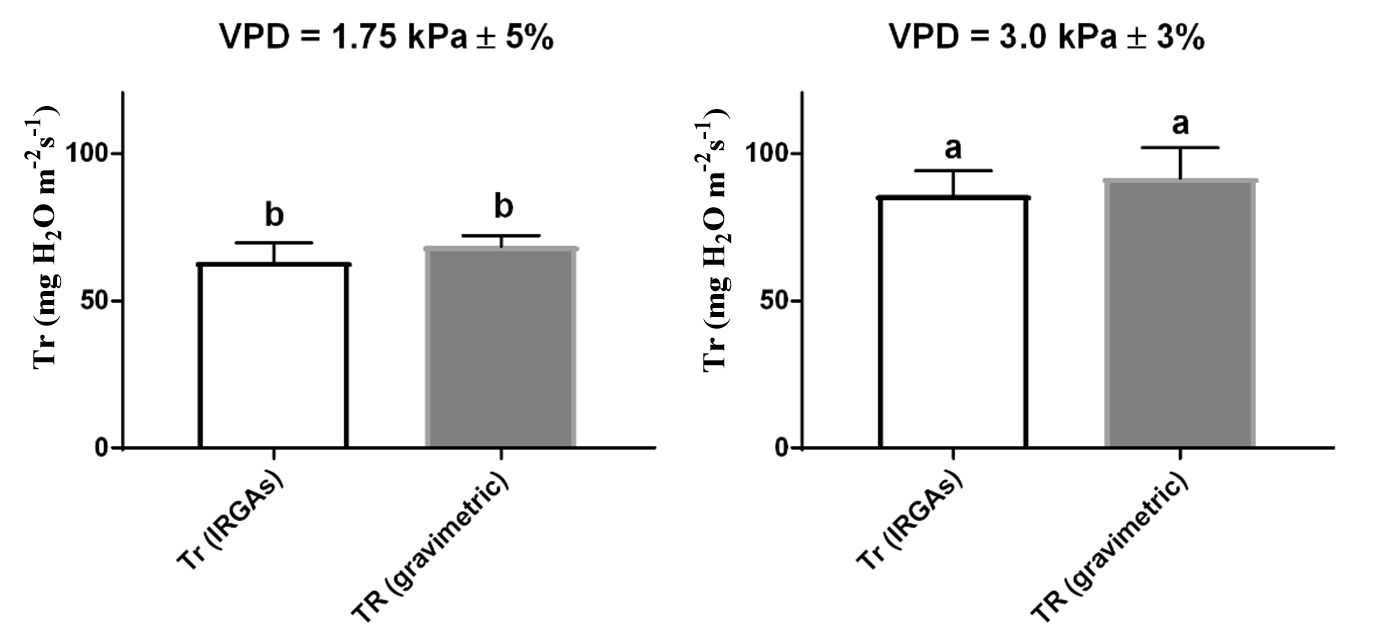


**Supplemental Figure 7.** Comparison of transpiration results using IRGAs and a gravimetric method in wheat Cadenza plants at 2 contrasting VPD. (Final table will include a comparison of all plants). The plants were watered to pot capacity. After 30 min, the pot was carefully covered with tape to avoid evaporation. The plant was moved from the greenhouse into the lab and the pot weighed. After that, the plant was placed in the chamber for ~20 min until a steady state VPD of 1.75 kPa was achieved. We measured Tr for 1h recording every 1 min. After that, the plant were removed for weighting. The same plant was placed in the chamber and same procedure were done, this time at 3 kPa VPD. The experiment were repeated with 5 plants. Values with different letters are significantly (P < 0.05) different.


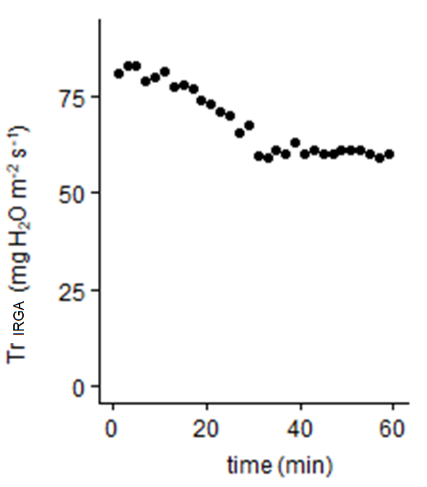


**Supplemental Fig. 8**. Reduction of the transpiration rates (Tr) after spraying [ABA] on an individual plant of wild type Steptoe exposed to 2.5 kPa VPD.


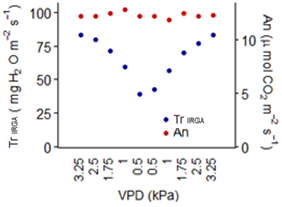


**Supplemental Fig. 9.** Test of hysteresis with a wheat plant (cv. Cadenza) in the system. The plant is sequentially exposed to decreases and increases of VPD over time.
